# Outcomes and prognostic factors in patients with locally advanced cervical cancer treated with concurrent chemoradiotherapy

**DOI:** 10.1186/s13014-022-02115-1

**Published:** 2022-08-17

**Authors:** Jing Liu, Guyu Tang, Qin Zhou, Weilu Kuang

**Affiliations:** 1 Department of Oncology, Xiangya Hospital, Central South University, No. 87 Xiangya Road, Kaifu District, Changsha, 410008 Hunan People’s Republic of China; 2 Department of Urology, Xiangya Hospital, Central South University, No. 87 Xiangya Road, Kaifu District, Changsha, 410008 Hunan People’s Republic of China

**Keywords:** Locally advanced cervical cancer, Concurrent chemoradiotherapy, Recurrence or metastasis, Prognostic factors

## Abstract

**Background:**

To investigate the prognostic factors affecting long-term survival in locally advanced cervical cancer (LACC) patients treated with concurrent chemoradiotherapy (CCRT).

**Methods:**

We retrospectively analyzed 192 naive LACC (stage IIB–IVA) patients who underwent intensity-modulated radiotherapy (IMRT) with concurrent platinum-based chemotherapy in Xiangya Hospital from January 2014 to June 2017. The clinicopathological factors of all patients were collected. To explore the relationship between factors and prognosis, survival rates were estimated by the Kaplan–Meier method. Univariate and multivariate Cox proportional hazards models were used to evaluate the effect of various factors on overall survival (OS) and progression-free survival (PFS). The nomogram and calibration curves were generated on the basis of survival analysis.

**Results:**

The median follow-up time was 39.5 months. There-year rates of OS and PFS were 89.1% and 82.8%. LACC patients with non-squamous cell carcinoma [NSCC, including adenocarcinoma or adenosquamous carcinoma (AC/ASC)], advanced stage (IIIA-IVA), initially positive lymph node (pelvic or para-aortic lymph node, PLN/PALN), and a lower pretreatment hemoglobin (HGB) level (< 126 g/L) had lower survival rates. In univariate analysis, patients with NSCC, advanced stage, PLN or PALN metastasis had worse OS. Patients with NSCC, advanced stage, PLN or PALN metastasis, and a lower pretreatment HGB level had worse PFS. In multivariate analysis, NSCC and PALN metastasis were independent prognostic parameters of OS. NSCC, PALN metastasis and a lower pretreatment HGB level were independent prognostic parameters of PFS.

**Conclusions:**

NSCC and PALN metastasis were poor prognostic factors of OS and PFS, a lower pretreatment HGB level was an independent prognostic factor of PFS in LACC patients treated with CCRT.

## Introduction

Cervical cancer ranks fourth in global female incidence and mortality, with approximately 604,000 new diagnoses and 342,000 deaths in 2020 [1]. For patients with locally advanced cervical cancer (LACC), defined as stage IIB-IVA based on International Federation of Gynecology and Obstetrics (FIGO) staging system (version 2018), definitive platinum-based concurrent chemoradiotherapy (CCRT) has been the standard treatment [2]. Five clinical trials demonstrated 30%-35% reduction in risk of death for LACC patients treated by CCRT compared to radiotherapy (RT) alone [3–7]. The five-year overall survival (OS) rate for LACC patients is 70% or so after completion of synchronized chemoradiotherapy (CRT) [2]. However, approximately 35% of patients still experience disease progression after CRT, and the prognosis remains poor [8]. In addition to CRT, neoadjuvant chemotherapy followed by surgery represents an alternative approach. Although both treatments showed similar OS benefit, definitive CRT resulted in superior disease-free survival [9, 10]. To improve survival and prognosis, immunotherapy in recent years, especially immune checkpoint inhibitors, appears to be a promising therapeutic strategy that could be applied in the treatment of LACC patients [11].

Prognostic factors for LACC include race, age, stage, grade, histologic type, tumor volume, lymph node involvement and location, performance status, and the treatment received [2]. In addition to the above factors, novel prognostic biomarkers that can estimate condition, evaluate prognosis and guide therapy are required for patients with LACC. Several previous studies have shown that hematological indicators, especially hemoglobin (HGB), are prognostic indicators for patients with cervical cancer [12–14]. However, the impact of HGB levels on the prognosis of LACC patients treated with CCRT is still controversial [15].

In this retrospective study, disease was restaged according to the 2018 FIGO system, which has additional criteria related to lymph node metastasis (LNM). All patients received platinum-containing concurrent chemotherapy (CCT) with intensity-modulated radiotherapy (IMRT) plus brachytherapy (BCT), and we reviewed more factors. We sought to explore prognostic factors in LACC patients undergoing CCRT treatment and to determine whether anemia truly affects patient survival. Another objective was to assess CCRT efficacy and to screen LACC patients who had a poor prognosis to guide further therapy in patients with high-risk factors.

## Materials and methods

### Research design

This study retrospectively included patients with LACC who underwent definitive IMRT and platinum-containing CCT between 1/1/2014 and 30/6/2017 in Xiangya Hospital, Central South University. Patients signed a written informed consent prior to therapy. The Ethics Committee in Xiangya Hospital approved this research (Ethical Review Number 201912525), which conformed to recognized standards of Declaration of Helsinki. The basic information of patients, tumor condition, examination results and therapeutic regimen were extracted from medical records, including the onset age, histological type, lymph node involvement, human papillomavirus (HPV) infection, number of childbirths and abortions, as well as regimen and dose of chemoradiation. Criteria for LNM were central necrosis or a short diameter ≥ 10 mm on computed tomography scans, heterogeneous enhanced signal intensity on magnetic resonance imaging, or increased fluorodeoxyglucose uptake in positron emission tomography scans. A cumulative irradiation dose of 45–50.4 Gy in 1.8–2 Gy/fraction, five fractions/week, was delivered to the planning target volume. The dose given to positive lymph nodes was increased to 54–60 Gy. For high dose rate (HDR) BCT, the prescribed dose was 30–36 Gy in 5–7 fractions to point A. Patients received concurrent weekly cisplatin/nedaplatin or platinum plus paclitaxel/docetaxel every three weeks.

### Patients

**Fig. 1:**
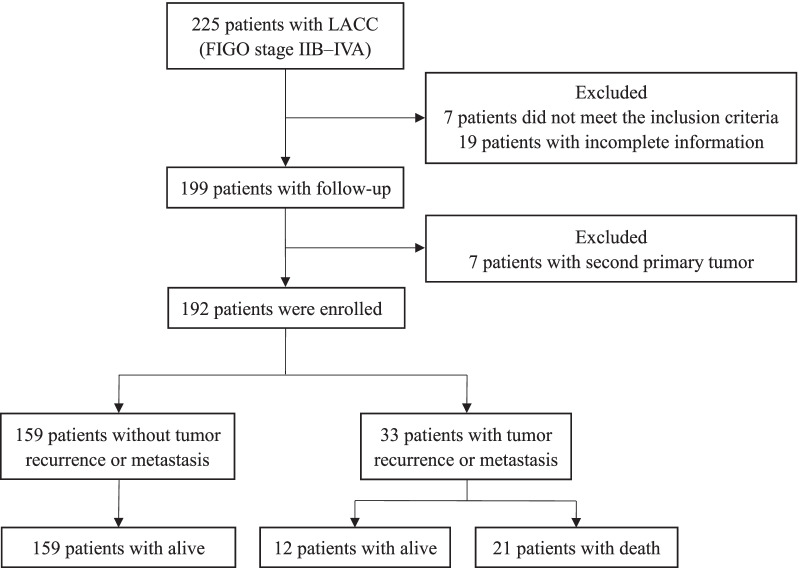
Flow chart outlining the patient-collection process

A flow chart outlining the patient-collection process is shown in Fig. 1. First, 225 LACC patients with stage IIB-IVA were retrospectively recruited in our study. The following inclusion criteria were adopted: (1) histological biopsy confirmed squamous cell carcinoma (SCC), adenocarcinoma (AC) or adenosquamous carcinoma (ASC) of the cervix; (2) Karnofsky Performance Scale score ≥ 70; (3) between the ages of 18 and 75 years; (4) initial patient without previous treatment; (5) completion of IMRT with platinum-containing CCT and HDR BCT on schedule. Consequently, we excluded 7 patients not meeting the inclusion criteria and 19 patients without complete clinical information. In addition, 7 patients with second primary malignancy detected during follow-up were excluded. Ultimately, 192 patients were enrolled in this study.

### Statistical analysis

The endpoints in this study were progression-free survival (PFS) and OS. PFS was calculated from initial date of CCRT to tumor progression or the last follow-up. OS was measured from initial date of CCRT to death or the last follow-up. SPSS software (version 25.0) and R software (version 4.1.0) were used for statistical analyses of this study. Survival rates were estimated by the Kaplan–Meier method, and curves were compared by the log-rank test. Univariate and multivariate Cox proportional hazards models were used to evaluate the influence of parameters on survival. Each parameter was assessed by univariate analysis. Age, histology, FIGO stage, lymph node involved, HPV infection, adjuvant chemotherapy (ACT) cycle and pretreatment HGB level were included for multivariate analysis to estimate hazard ratio and 95% confidence interval. Statistical significance was defined as *P* values < 0.05. On the basis of the results of survival analysis, the nomogram model was constructed using R software with the rms package. Calibration of the nomogram was performed by comparing the predicted probability with the actual probability of OS. The bootstrap method was used for internal validation to estimate the optimism of the model.

## Results

### Baseline characteristics of patients

Table 1 shows clinical data and characteristics of 192 LACC patients. The median age was 52 years, with a range from 32 to 73 years. Among all patients, 178 were diagnosed with SCC, 12 with AC, and 2 with ASC. Based on the 2018 FIGO staging system, 101 cases were restaged to stage IIIC. Eighty-six cases had no metastatic lymph nodes and 106 cases were diagnosed of lymph node metastases, of which 92 were positive for pelvic lymph node (PLN) only, and 14 were positive for PLN and para-aortic lymph node (PALN). We obtained pretreatment HGB levels for all patients and found a median HGB level of 126 g/L prior to initiation of treatment. All patients completed IMRT plus BCT and concurrent platinum-based chemotherapy as prescribed. Then, 162 patients received ACT, and 30 patients did not receive ACT.

**Table 1:** Clinical data and characteristics of LACC patients (n = 192)

Characteristic	No. of patients	Percentage (%)
Age		
< 52 y	87	45.3
≥ 52 y	105	54.7
Histology		
SCC	178	92.7
AC	12	6.3
ASC	2	1.0
FIGO stage		
IIB	68	35.4
IIIA	3	1.6
IIIB	11	5.7
IIIC	101	52.6
IVA	9	4.7
Lymph node metastasis		
Negative	86	44.8
PLN only	92	47.9
PLN and PALN	14	7.3
HPV infection		
Positive	114	59.4
Negative	78	40.6
Childbirth		
< 3	137	71.4
≥ 3	55	28.6
Abortion		
< 3	171	89.1
≥ 3	21	10.9
ACT		
Yes	162	84.4
No	30	15.6
Pretreatment HGB level (g/L)		
< 126	88	45.8
≥ 126	104	54.2

LACC: Locally advanced cervical cancer; SCC: squamous cell carcinoma; AC: adenocarcinoma; ASC: adenosquamous carcinoma; FIGO: International Federation of Gynecology and Obstetrics; PLN: pelvic lymph node; PALN: para-aortic lymph node; HPV: human papillomavirus; ACT: adjuvant chemotherapy; HGB: hemoglobin

### Kaplan–Meier survival analysis

**Fig. 2:**
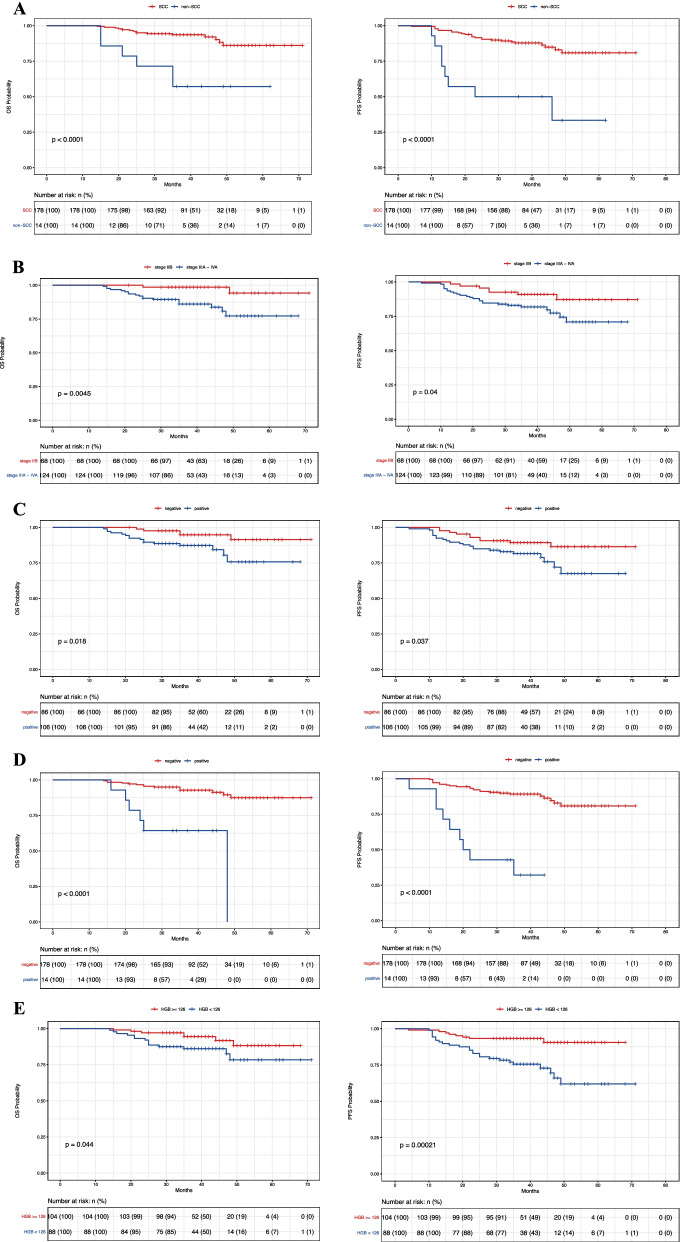
The OS and PFS rates according to Histology (**a**), FIGO Stage (**b**), PLN (**c**), PALN (**d**), Pretreatment HGB level (**e**). The differences in OS and PFS were estimated using the Kaplan–Meier method. OS: Overall survival; PFS: progression-free survival; SCC: squamous cell carcinoma; FIGO: International Federation of Gynecology and Obstetrics; PLN: pelvic lymph node; PALN: para-aortic lymph node; HGB: hemoglobin

192 LACC patients were followed up, with the first follow-up at 3 months after the end of CCRT and follow-up dates until December 2019. During follow-up (median 39.5 [14, 71] months), 33 patients (17.2%) experienced tumor recurrence or metastasis, and among them, 21 patients (10.9%) died (Fig. 1). The 3-year rates of PFS and OS for all patients were 82.8% and 89.1%. Figure 2 shows Kaplan–Meier analysis results. OS rates between the SCC and non-SCC (AC/ASC) groups were 91.6% versus 57.1%, and PFS rates were 86.0% versus 42.9%, respectively (Fig. 2a). Compared to stage IIB, patients in stage IIIA- IVA had poorer OS and PFS (Fig. 2b). OS and PFS rates in patients with negative and positive PLNs were 94.2% versus 82.6% and 88.4% versus 75.0%, respectively (Fig. 2c). OS and PFS rates in patients with negative and positive PALNs were 91.6% versus 57.1% and 86.5% versus 35.7%, respectively (Fig. 2d). OS and PFS rates in patients with pretreatment HGB levels ≥ 126 g/L and < 126 g/L were 93.3% versus 84.1% and 92.3% versus 71.6%, respectively (Fig. 2e).

### Univariate and multivariate Cox analyses of survival

**Fig. 3:**
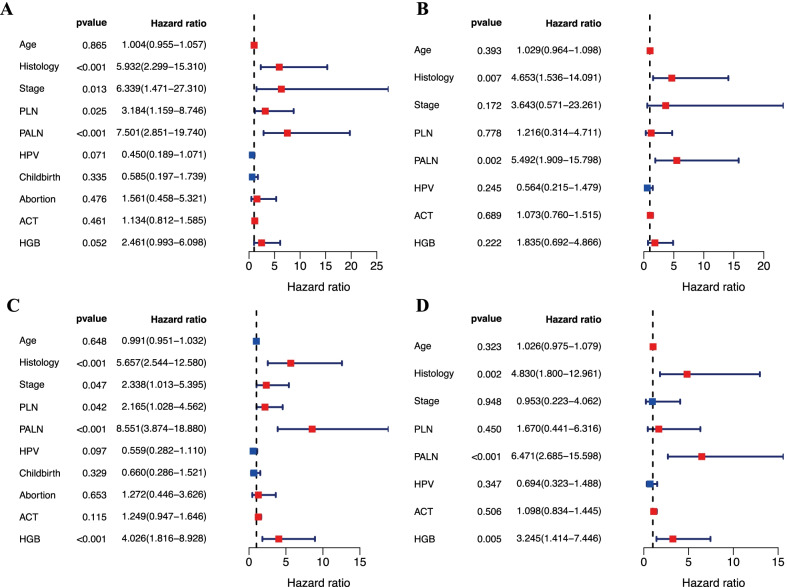
Forest plots based on univariate and multivariable Cox analyses of OS (**a**, **b**) and PFS (**c**, **d**) in patients with LACC. OS: Overall survival; PFS: progression-free survival; PLN: pelvic lymph node; PALN: para-aortic lymph node; HPV: human papillomavirus; ACT: adjuvant chemotherapy; HGB: hemoglobin

The univariate and multivariate analyses for OS were summarized in Table 2. Non-squamous cell carcinoma (NSCC, AC or ASC), advanced stage (IIIA-IVA), positive PLN and PALN were significant prognostic parameters of OS (Fig. 3a). NSCC (*P* = 0.007) and PALN metastasis (*P* = 0.002) were independent factors of OS (Fig. 3b). The univariate and multivariate analyses for PFS were summarized in Table 3. NSCC (AC or ASC), advanced stage (IIIA-IVA), positive PLN and PALN, and a lower pretreatment HGB level (< 126 g/L) were significant prognostic parameters of PFS (Fig. 3c). NSCC (*P* = 0.002), PALN metastasis (*P* < 0.001), and a lower pretreatment HGB level (*P* = 0.005) were independent factors of PFS (Fig. 3d).

**Table 2:** Prognostic effect of variables on overall survival

Characteristic	Univariate analysis		Multivariate analysis	
	HR (95% CI)	*P* value	HR (95% CI)	*P* value
Age (≥ 52 y vs. < 52 y)	1.004 (0.955–1.057)	0.865	1.029 (0.964–1.098)	0.393
Histology (AC/ASC vs. SCC)	**5.932 (2.299–15.310)**	< 0.001*	**4.653 (1.536–14.091)**	0.007*
FIGO stage (IIIA-IVA vs. IIB)	**6.339 (1.471–27.310)**	0.013*	3.643 (0.571–23.261)	0.172
PLN (Positive vs. Negative)	**3.184 (1.159–8.746)**	0.025*	1.216 (0.314–4.711)	0.778
PALN (Positive vs. Negative)	**7.501 (2.851–19.740)**	< 0.001*	**5.492 (1.909–15.798)**	0.002*
HPV infection (Positive vs. Negative)	0.450 (0.189–1.071)	0.071	0.564 (0.215–1.479)	0.245
Childbirth (≥ 3 vs. < 3)	0.585 (0.197–1.739)	0.335		
Abortion (≥ 3 vs. < 3)	1.561 (0.458–5.321)	0.476		
ACT (Yes vs. No)	1.134 (0.812–1.585)	0.461	1.073 (0.760–1.515)	0.689
Pretreatment HGB level (< 126 g/L vs. ≥ 126 g/L)	2.461 (0.993–6.098)	0.052	1.835 (0.692–4.866)	0.222

HR: Hazard ratio; CI: confidence interval; SCC: squamous cell carcinoma; AC: adenocarcinoma; ASC: adenosquamous carcinoma; FIGO: International Federation of Gynecology and Obstetrics; PLN: pelvic lymph node; PALN: para-aortic lymph node; HPV: human papillomavirus; ACT: adjuvant chemotherapy; HGB: hemoglobin

Bold indicates significant values of *P < 0.05

**Table 3:** Prognostic effect of variables on progression-free survival

Characteristic	Univariate analysis		Multivariate analysis	
	HR (95% CI)	*P* value	HR (95% CI)	*P* value
Age (≥ 52 y vs. < 52 y)	0.991 (0.951–1.032)	0.648	1.026 (0.975–1.079)	0.323
Histology (AC/ASC vs. SCC)	**5.657 (2.544–12.580)**	< 0.001*	**4.830 (1.800–12.961)**	0.002*
FIGO stage (IIIA-IVA vs. IIB)	**2.338 (1.013–5.395)**	0.047*	0.953 (0.223–4.062)	0.948
PLN (Positive vs. Negative)	**2.165 (1.028–4.562)**	0.042*	1.670 (0.441–6.316)	0.450
PALN (Positive vs. Negative)	**8.551 (3.874–18.880)**	< 0.001*	**6.471 (2.685–15.598)**	< 0.001*
HPV infection (Positive vs. Negative)	0.559 (0.282–1.110)	0.097	0.694 (0.323–1.488)	0.347
Childbirth (≥ 3 vs. < 3)	0.660 (0.286–1.521)	0.329		
Abortion (≥ 3 vs. < 3)	1.272 (0.446–3.626)	0.653		
ACT (Yes vs. No)	1.249 (0.947–1.646)	0.115	1.098 (0.834–1.445)	0.506
Pretreatment HGB level (< 126 g/L vs. ≥ 126 g/L)	**4.026 (1.816–8.928)**	< 0.001*	**3.245 (1.414–7.446)**	0.005*

HR: Hazard ratio; CI: confidence interval; SCC: squamous cell carcinoma; AC: adenocarcinoma; ASC: adenosquamous carcinoma; FIGO: International Federation of Gynecology and Obstetrics; PLN: pelvic lymph node; PALN: para-aortic lymph node; HPV: human papillomavirus; ACT: adjuvant chemotherapy; HGB: hemoglobin

Bold indicates significant values of *P < 0.05

### Nomogram model and verification for survival prediction

**Fig. 4:**
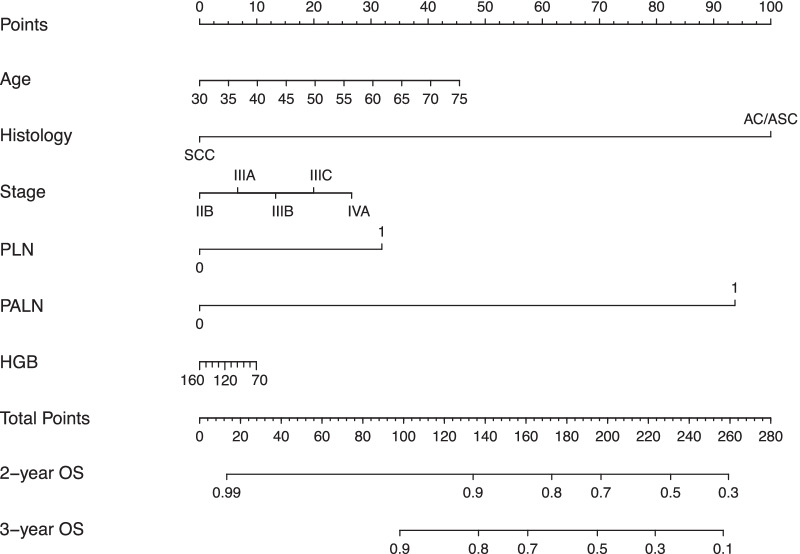
Nomogram model for LACC patients to predict the 2- and 3-year OS. PLN indicates positive (1) or negative (0) pelvic lymph nodes. PALN indicates positive (1) or negative (0) para-aortic lymph nodes. To use, find patient’s age on age axis, then draw straight line upward to points axis to determine how many points patient receives for age and do this again for other variable axes. The total points predicted on the bottom scale was calculated by summing all points on the scale for each variable to estimate the probabilities of 2-year and 3-year OS rates by plotting a vertical line. OS: Overall survival; SCC: squamous cell carcinoma; AC: adenocarcinoma; ASC: adenosquamous carcinoma; PLN: pelvic lymph node; PALN: para-aortic lymph node; HGB: hemoglobin

**Fig. 5:**
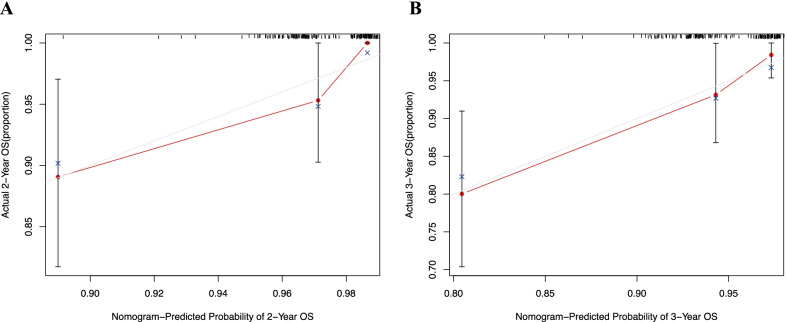
The calibration curves for predicting OS for LACC patients at 2 years (**a**) and 3 years (**b**) in the verification. Calibration of the nomogram for OS by comparing the predicted survival (plotted on the X-axis) with the actual survival (plotted on the Y-axis). OS: Overall survival

A nomogram model containing predictive variables was developed to predict the survival rates of LACC patients. The concordance index of the OS nomogram was 0.79. Figure 4 can be used to compute the probability of 2-year OS and 3-year OS. The sum of the points of each variable was plotted on the total points axis, and the estimated probability of survival could be obtained by drawing a vertical line from the axis of total points straight down to the survival-probability axis. Histology and PALN metastasis were important predictors of OS at two and three years. Calibration curves showed good conformity between actual and predicted probability of OS (Fig. 5).

## Discussion

Concomitant cisplatin-based CRT has been the mainstay of treatment for women with cervical carcinoma according to five large randomized controlled trials (RCTs) since 1999 [3–7]. A meta-analysis of 13 trials showed a 6% increase in absolute survival benefit at five years with CRT compared to RT alone for cervical cancer patients [8]. In brief, CCRT can reduce the risk of disease progression and improve survival for LACC patients. However, the benefit of CCRT on survival was thought to decrease with increasing staging [8]. For patients with LACC, FIGO staging is not sufficient to fully evaluate the therapeutic effect and judge the prognosis.

In our study, the retrospective analysis was performed on 192 LACC patients (FIGO staging IIB-IVA, 2018 edition) who received unified therapy, including IMRT combined with concurrent platinum-based chemotherapy plus BCT. The five trials above showed a local relapse rate of 19%-20% after conventional radiation [3–7]. Compared to conventional RT, IMRT shows a more conformal dose distribution, resulting in a higher targeted dose and a lower normal organ dose. This study demonstrated a recurrence and metastasis rate of 17.2% and a death rate of 10.9% after 3 years of follow-up. Although standard CCRT could generally achieve good results in patients with LACC, some patients still experienced recurrence or metastasis, affecting the long-term prognosis. Therefore, we evaluated prognostic parameters affecting the survival for LACC patients, which could help to assess therapeutic effect and guide individual treatment after CCRT.

The most common histopathology for carcinoma in cervix is SCC (about 80% of cases), and its incidence and mortality rate have declined, which is largely attributed to the effectiveness of screening programs [16, 17]. Nevertheless, the incidence of AC/ASC in cervical cancer has increased, especially among young women [17–19]. Whether different pathological subtypes of LACC have different outcomes and survival remains controversial. Katanyoo et al. found that AC showed a poorer response rate than SCC for RT/CCRT and a longer time to achieve complete response for LACC patients, while there was no difference in 5-year OS between the two types, and the pathological type was not a determinant of survival outcomes [17]. In contrast, Rose et al. [20] found that locally advanced cervical AC/ASC was related to poorer survival when undergoing radiation alone but similar survival when undergoing concurrent cisplatin compared to SCC. However, another study showed that patients with AC had poorer OS than SCC both before and after the introduction of CCRT [21]. In our study, survival rates were significantly different between SCC and AC/ASC groups, and the histologic subtype was a prognostic parameter of OS and PFS. However, the reasons for different prognoses between SCC and AC/ASC has yet to be explored, and prospective researches are necessary to explore histology-specific therapy.

One major change in this 2018 FIGO staging system compared to the prior 2014 version is the addition of IIIC staging regarding imaging or pathological lymph node involvement. PLN positivity alone is identified as stage IIIC1 and PALN metastasis as stage IIIC2 [22]. This new staging system highlights the significance of LNM as a prognostic indicator in carcinoma of uterine cervix. A retrospective observational study found that the survival of patients with IIIC1 disease was superior to IIIA-B disease. Additionally, the 5-year survival rates for stage IIIC1 patients significantly differed according to T-staging (T1: 74.8%, T2: 58.7%, T3: 39.3%), which demonstrated that stage IIIC1 reflects tumor heterogeneity and that the survival of patients with IIIC1 is related to local tumor factors [23]. The study by Chen et al. [14] revealed that initial LNM was an indicator of poor prognosis for LACC disease. Yamashita et al. found that PLN and PALN status significantly affected survival and that PALN metastasis was the most significant prognostic factor for LACC patients [24]. Another study found that common iliac LNM and bilateral pelvic LNM were risk factors for distant metastasis in cervical cancer patients received IMRT [25]. In our research, the most common substage was stage IIIC (n = 101, 52.6%), followed by stage IIB (n = 68, 35.4%). Because of the low number of stage IIIA, IIIB and IVA cases (12.0% in total), this study classified stages IIIA-IVA into one group (advanced stages) and stage IIB into another group. The univariate analysis suggested significant differences in OS and PFS between two groups, while the multivariate analysis suggested that FIGO staging was not an independent indicator affecting prognosis. Overall, the survival rate and prognosis cannot be evaluated by the FIGO stage alone. Our study demonstrated that PLN and PALN metastases were significantly related to worse OS and PFS, while only PALN metastasis was an independent prognostic indicator affecting survival. Our results are largely consistent with the previously mentioned reports, while the need for prophylactic extended field radiation in node-positive patients needs to be further explored.

For patients with cervical carcinoma, anemia or a low HGB level has been considered in most studies as an indicator of poor prognosis which can affect the efficacy of RT [15]. A study in 1965 first published findings on the relationship between HGB and cervical cancer prognosis. The results suggested that OS was lower in patients with pretreatment HGB levels < 110 g/L and that a lower HGB resulted in a higher rate of treatment failure [12]. Haensgen et al. found that pretreatment HGB was an independent prognostic indicator in LACC patients [13]. Moreover, Chen et al. demonstrated that a lower HGB level before treatment was associated with poorer local control [14]. However, a retrospective cohort study concluded that anemia was not an independent factor for cervical cancer relapse after RT [15]. Therefore, the impact of HGB levels on prognosis in patients with carcinoma of cervix is controversial. Based on our 3-year surveillance data, LACC patients with lower pretreatment HGB levels had worse OS and PFS, and the pretreatment HGB level was an independent factor of PFS. “Hypoxic radioresistance” is commonly used to explain the correlation between anemia and cancer progression, and several interventions have been investigated to overcome hypoxia-induced radiation resistance, including hyperbaric oxygenation, erythropoietin injection, blood transfusion and drug therapy [15, 26]. A study presented the first expert consensus guideline which recommended a minimum HGB of 90 g/L as a transfusion target to balance tumor radiation sensitivity with rational allocation of inadequate blood resources in cervical cancer patients undergoing RT [27]. On the whole, future clinical RCTs are required to evaluate potential interrelationships between anemia, tumor hypoxia, angiogenesis and the effectiveness of RT.

Moore et al. found that for LACC patients > 50 years, the risk of death due to all-cause mortality increased by 2% for each additional year, while age did not correlate significantly with disease-specific PFS and OS [28]. Our study used the median age (52 years) as a cutoff point, and our results similarly showed no significant difference for OS and PFS.

Several studies have revealed that low initial HPV viral load or HPV-negativity indicated a poorer prognosis for cervical cancer patients undergoing RT [29–31], and persistent HPV DNA was associated with local relapse after RT [32]. Conversely, other studies have shown that the HPV viral DNA copy number could not predict survival for patients with cervical carcinoma [33, 34]. This study found no significant relationship between initially positive HPV and prognosis in LACC patients. Of note, HPV detection is commonly recommended as part of follow-up examinations, which may be useful in treatment effectiveness evaluation.

Previous studies have suggested that women with none or numerous pregnancies had poorer survival than others with a few pregnancies before developing cervical carcinoma [35–37]. In our series, the effects of the number of childbirths and abortions on OS and PFS were not significant, but LACC patients with ≥ 3 abortions prior to CCRT had a relatively poor prognosis.

A systematic review showed greater benefits for trials in which ACT was administered after CRT, with a 19% absolute improvement at 5 years [8]. A phase III, open-label RCT demonstrated better survival in patients undergoing gemcitabine and cisplatin chemoradiation followed by brachytherapy and ACT compared to standard CCRT treatment [38]. Nevertheless, Tangjitgamol et al. [39] concluded insufficient evidence to support ACT after CCRT with limited data from two RCTs. Our data showed no significant difference of OS and PFS for LACC patients who received ACT or not after CCRT. Although several studies have suggested that ACT may benefit LACC patients, large prospective RCTs are still needed to assess the role of different ACT regimens and cycle numbers to further explore the impact of ACT on survival.

Several limitations also existed in our study. First, the number of LACC patients enrolled was limited, and large multicenter prospective researches are necessary to further clarify prognostic factors. Second, no external validation of the nomogram was performed using an independent group of patients. Moreover, long-term follow-up results, especially outcomes and toxicity in patients undergoing CCRT plus ACT, need to be analyzed in detail to assess the value of ACT and develop individualized treatment regimens.

## Conclusions

Our study demonstrated that NSCC (AC or ASC) and PALN metastasis were significantly related to decreased OS and PFS for LACC patients undergoing CCRT. A lower pretreatment HGB level was an independent indicator of PFS which may function as a prognostic biomarker.

## Data Availability

The datasets used and/or analysed during the current study are available from the corresponding author on reasonable request.

## Abbreviations

AC: Adenocarcinoma
ACT: Adjuvant chemotherapy
ASC: Adenosquamous carcinoma
BCT: Brachytherapy
CCRT: Concurrent chemoradiotherapy
CCT: Concurrent chemotherapy
CRT: Chemoradiotherapy
FIGO: International Federation of Gynecology and Obstetrics
HDR: High dose rate
HGB: Hemoglobin
HPV: Human papillomavirus
IMRT: Intensity-modulated radiotherapy
LACC: Locally advanced cervical cancer
LNM: Lymph node metastasis
NSCC: Non-squamous cell carcinoma
OS: Overall survival
PALN: Para-aortic lymph node
PFS: Progression-free survival
PLN: Pelvic lymph node
RCTs: Randomized controlled trials
RT: Radiotherapy
SCC: Squamous cell carcinoma
